# Xanthogranulomatous Pyelonephritis in a male child with renal vein thrombus extending into the inferior vena cava: a Case Report

**DOI:** 10.1186/1471-2431-10-47

**Published:** 2010-07-06

**Authors:** Geetanjali Gupta, Reecha Singh, Dhananjay S Kotasthane, Vaishali D Kotasthane, Shailesh Kumar

**Affiliations:** 1 Department of Pathology, Mahatma Gandhi Medical College & Research Institute, Pillaiyarkuppam, Puducherry, India; 2 Department of Microbiology, Mahatma Gandhi Medical College & Research Institute, Pillaiyarkuppam, Puducherry, India

## Abstract

**Background:**

We present a case of Xanthogranulomatous pyelonephritis (XGPN) in a male child with renal vein thrombus extending into the inferior vena cava. This is a rare presentation. XGPN is a rare type of renal infection characterised by granulomatous inflammation with giant cells and foamy histiocytes. The peak incidence is in the sixth to seventh decade with a female predominance. XGPN is rare in children.

**Case presentation:**

An 11 year old male child presented with a history of high grade fever and chills, right flank pain and progressive pyuria for two months. He had a history of vesical calculus for which he was operated four years back. In our case, a subcapsular right nephrectomy was performed. The surgical specimens were formalin fixed and paraffin embedded. The sections were stained with routine Hematoxylin & Eosin stain. Grossly; the kidney was enlarged with adherent capsule and thickening of the perinephric tissue. The pelvicalyceal system was dilated and was filled with a cast of pus. Histological evaluation revealed diffuse necrosis of the renal parenchyma and perinephric fat. Neutrophils, plasma cells, sheets of foamy macrophages and occasional multinucleate giant cells were seen. The renal vein was partially occluded by an inflammatory thrombus with fibrin, platelets and mixed inflammatory cells. The thrombus was focally adherent to the vein wall with organization.

**Conclusions:**

The clinical presentation and the macroscopic aspect, together with the histological pattern, the cytological characteristics addressed the diagnosis towards XGPN with a vena caval thrombus. Our case illustrates that the diagnosis of XGPN should be considered even in paediatric age group when renal vein and vena caval thrombi are present.

## Background

Xanthogranulomatous pyelonephritis (XGPN) in a male child with a renal vein thrombus extending into the inferior vena cava is an extremely rare presentation. XGPN is a chronic renal infection characterised by destruction and replacement of the renal parenchyma with sheets of lipid laden macrophages, admixed acute and chronic inflammatory cells and frequent abscess formation [1]. The peak incidence is in the sixth to seventh decade with a female predominance. XGPN is rare in children [2]. We report a case of XGPN in a male child presenting with a vena caval thrombus.

## Case presentation

An 11 year old male child presented with a history of high grade fever with chills, right flank pain and progressive pyuria for two months. He had a prior history of surgery for vesical calculus and anterior urethral calculus for which he underwent open cystolithotomy and urethrolithotomy four years back. The chemical nature of the stone was calcium oxalate dihydrate. Medical work-up did not reveal any cause for the calculus.

On examination, the child was emaciated, febrile with pallor. There was diffuse tenderness in the right lumbar region with renal angle tenderness. X-ray showed radio-opaque shadow in the right lumbar region measuring 2 × 2 cm. Abdominal computerised tomography (CT) revealed a dilated pelvicalyceal system with necrotic debris at the lower pole of right kidney and a thrombus in the renal vein extending into the infrahepatic vena cava. Pyonephrosis, parenchymal calcification along with marked retroperitoneal and porta hepatis lymphadenopathy were also seen on the CT. Blood urea and serum creatinine were within normal limits. Gram negative bacilli, *Pseudomonas aeruginosa *were isolated from thick pus aspirated from the right renal pelvis. The urine culture was sterile.

Open nephrostomy was done which drained only pus and no urinary drainage. Following a renal biopsy diagnosed as chronic pyelonephritis (end-stage changes), the patient underwent subcapsular right nephrectomy. Kidney exposure was accomplished through the flank extraperitoneally using a 12th rib cutting incision. There were dense adhesions of the kidney and Gerota's fascia with the surrounding structures. It was decided to do a subcapsular nephrectomy. When the kidney was reached, the capsule was incised and is freed from the underlying cortex. The capsule was stripped from the surface of the kidney, and an incision was made all around the capsule where it was attached to the hilum. The vessels were protected by placing a finger in front of the pedicle while cutting the capsule. The adhesions present around the pedicle were meticulously separated out. To avoid damage to the duodenum or major vessels, pieces of adherent capsule were left behind. The renal vein appeared enlarged with multiple venous collaterals. The renal vein control was obtained proximally and it was longitudinally incised and the thrombus was delivered out in its entirety. After ligation and cutting of the pedicle, the ureter was identified and cut, and the distal end was ligated.

## Methods

The surgical specimens were formalin fixed and paraffin embedded. The sections were stained with routine Hematoxylin &Eosin stain. Written consent was obtained from the patient for publication of patient details.

## Results

### Gross appearance

**Figure 1:**
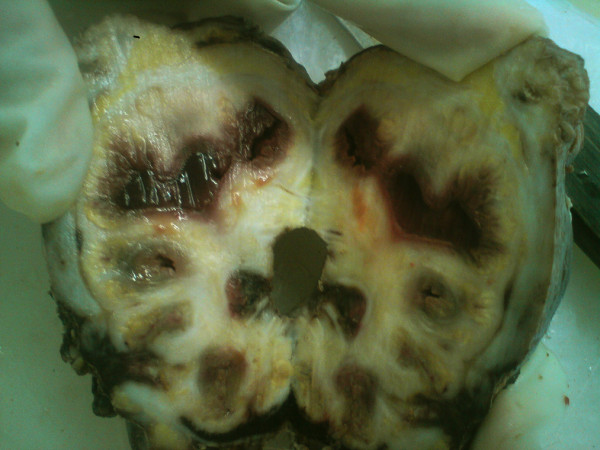
**Gross photograph**. Specimen showing dilated calyces filled with purulent material and adjacent yellow areas.

Grossly, the sub capsular nephrectomy specimen weighed 155 gms and measured 10 × 6.5 × 5 cm. The cut surface showed a dilated pelvicalyceal system filled with a cast of pus. Adjacent to the pelvis and calyces, yellow areas were seen. The renal cortex was thinned out. The capsule and perinephric tissue were thickened with adhesions (Figure 1).

### Microscopic appearance

**Figure 2:**
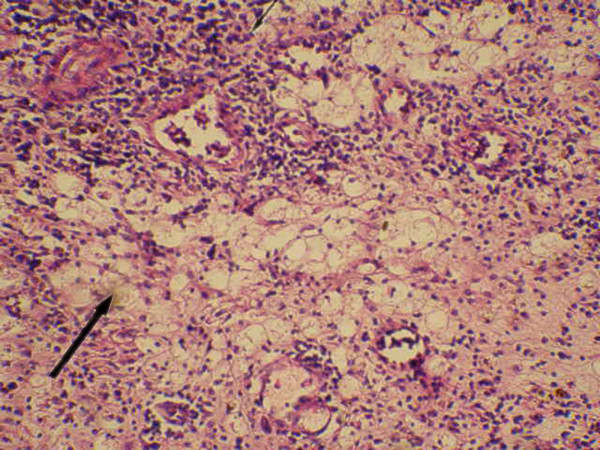
**Hematoxylin and Eosin staining (200×)**. Showing sheets of fat laden macrophages and occasional multinucleate giant cells. Thick arrow indicates sheets of fat laden macrophages and thin arrow indicates dense chronic inflammatory cells.

Histological evaluation revealed diffuse necrosis of the renal parenchyma and perinephric fat. Neutrophils, plasma cells, sheets of foamy macrophages and occasional multinucleate giant cells were seen (Figure 2). The renal vein was partially occluded by an inflammatory thrombus with fibrin, platelets and mixed inflammatory cells. The thrombus was focally adherent to the vein wall with thrombus. Adjacent areas showed histological features of chronic pyelonephritis.

The clinical presentation and the macroscopic aspect, together with the histological pattern, the cytological characteristics addressed the diagnosis towards XGPN with a vena caval thrombus.

## Discussion

Xanthogranulomatous pyelonephritis (XGPN) is a severe, atypical chronic renal parenchymal infection accounting for 6/1000 surgically proved cases of chronic pyelonephritis [2]. Women are affected more frequently than men with a peak incidence in the sixth to seventh decades [3].XGPN is rare in children. In our case the patient was an 11 year old child. It has been suggested that the paediatric variant of XGPN differs from that of adults and, furthermore, sexual and racial differences in paediatric disease expression may exist [2]. Changes of XGPN have been described in kidneys destroyed as a result of pyonephrosis, renal cell carcinoma, transitional cell carcinoma and rarely a renal cyst [3]. Several interrelated etiological factors are thought to be responsible for the pathogenesis of XGPN. They include calculus or non calculus urinary obstruction, ineffectively treated urosepsis, chronic renal ischemia causing localised alteration in renal metabolism, lymphatic obstruction, alteration in lipid metabolism and an altered immune response [4]. In our case the etiological factor was a vesical calculus. The extent of the pathologic process within the affected kidney varies. XGPN is a chronic renal infection characterized by destruction and replacement of the renal parenchyma with sheets of lipid laden macrophages, admixed acute and chronic inflammatory cells, and frequent abscess formation [5] as in our case. In our patient acute inflammation is believed to have caused thrombosis of the renal vein with extension into the vena cava. A variety of conditions, particularly nephrotic syndrome, can lead to renal vein thrombosis (RVT). However, the exact incidence of RVT is not well known, because many patients remain asymptomatic [6]. Two forms of XGPN have been described, a diffuse or global form (83-90%) as in our case, and a focal form (10-17%) [3]. In the rare localised form, the lesion can be confined to one or other pole. More commonly it is a diffuse process involving whole kidney leading to reduced renal function. XGPN has been termed the great imitator because it may be misdiagnosed as a renal neoplasm especially if the lesion is focal. The most common presenting features are intermittent high grade fever, flank pain, loin pain, hematuria and vague gastrointestinal symptoms. Proteinuria and pyuria are frequent. Similar symptoms were found in our case. The organisms isolated on urine culture are Proteus mirabilis, Escherichia coli, Staphylococcus aureus, Klebsiella, Pseudomonas and Enterobacter species. Urine culture may show negative results in 30-39% of patients despite positive results from kidney [3]. In our case *Pseudomonas aeruginosa *was detected in pus culture while the urine culture was sterile. Mostly one kidney is affected. In a few cases contra lateral kidney is enlarged due to compensatory hypertrophy. XGPN has been described in three stages. The lesion is confined to kidney in stage I as in our case, extends to Gerota's space in stage II, to the perinephric space and other retroperitoneal structures in stage III.^3 ^Treatment of XGPN is partial or total nephrectomy [7,8].

## Conclusion

XGPN is a common histological variant of surgically managed end stage pyelonephritis. In paediatric patients with this process clinical and pathological findings are similar to those in adults. Pathogenesis and, thus, time to establishment of this process have not been clearly defined and may be of a shorter interval than currently believed. Given an appropriate clinical and radiographic presentation, and without regard to patient age or duration of symptoms, the diagnosis of XGPN should be made prospectively. Our case illustrates that the diagnosis of XGPN should be considered even in paediatric age group when renal vein and vena caval thrombi are present.

## Competing interests

The authors declare that they have no competing interests.

## Authors' contributions

GG drafted the manuscript, carried out the literature search and prepared the illustrations. RS conceived the idea of the study, helped to draft the manuscript and prepare the illustrations. DSK was responsible for overall coordination and final proofreading of the manuscript. VDK helped to draft the manuscript and acquire histological images for illustration. SK did the final proofreading of the manuscript. All the above mentioned authors read and approved the final manuscript.

## Pre-publication history

The pre-publication history for this paper can be accessed here:

http://www.biomedcentral.com/1471-2431/10/47/prepub
